# The minimal incubation period from the onset of Barrett's oesophagus to symptomatic adenocarcinoma

**DOI:** 10.1038/bjc.2011.214

**Published:** 2011-06-14

**Authors:** C M den Hoed, M van Blankenstein, J Dees, E J Kuipers

**Affiliations:** 1 Department of Gastroenterology and Hepatology, Erasmus Medical Centre, Room Ba393, PO Box 2040, 3000 CA Rotterdam, The Netherlands; 2 Department of Internal Medicine, Erasmus Medical Centre, PO Box 2040, 3000 CA Rotterdam, The Netherlands

**Keywords:** Barrett's oesophagus, epidemiology, oesophageal adenocarcinoma, dysplasia, survival

## Abstract

**Background:**

The interval between the onset of Barrett's oesophagus (BO) and oesophageal adenocarcinoma (OAC) can be termed the incubation period. However, the unrecorded onset of BO precludes its direct observation.

**Methods:**

Determining the range of intervals between BO diagnosis and OAC within the longest observational BO follow-up study. Exclusion criteria were presence of high-grade dysplasia (HGD) or OAC at baseline, death within <2 years of BO diagnosis, oesophagectomy without HGD/OAC and loss to follow-up. A total of 133 patients (M/F 73/60) were taken into account.

**Results:**

In 1967 person years of follow-up there were 13 cases of HGD/OAC, (0.66% p.a.; 95% CI 0.58–0.74), 96 patients died without HGD/OAC and 24 survived without HGD/OAC. The mean intervals between BO diagnosis and either HGD/OAC, death or end of follow-up were 10.8, 12.6 and 25.5 years, respectively, and the mean ages at endpoint were 72.5, 80.0 and 68.3 years, respectively. The survivors without HGD/OAC had a lower age at BO diagnosis (mean 42.8 *vs* 61.2 and 67.4 years, *P*<0.001). Baseline presence of low-grade dysplasia was associated with progression to HGD/OAC (log rank *P*<0.001).

**Conclusion:**

The Rotterdam BO follow-up cohort revealed a long incubation period between onset of BO and development of HGD/OAC, in patients without HGD/OAC at baseline as illustrated by 24 patients diagnosed with BO at a young age and followed for a mean period of 25.5 years. Their tumour-free survival established a minimum incubation period, suggesting a true incubation period of three decades or more.

Oesophageal cancer is currently the sixth leading cause of cancer-related death worldwide. Since the 1980s the incidence of oesophageal cancer has been rising by about 4–10% annually. (Pace *et al*, 2007; Odze, 2008). In the United States, SEER data over the period 1973–2004 demonstrated a rising oesophageal cancer incidence of 45% in Caucasian males and 3.6% in Caucasian females (Brown *et al*, 2008). These rises solely resulted from dramatic increases in the incidence of oesophageal adenocarcinoma (OAC) of 463% in males and 335% in females, as the squamous cell carcinoma incidence by sex simultaneously fell by 50% and 29% respectively (Brown *et al*, 2008). In The Netherlands, over the 15-year period from 1989 to 2003, the OAC incidence rose annually by 7.2% in males and 3.5% in females (van Blankenstein *et al*, 2007b). There can be little doubt about the involvement of the increasing incidence of Barrett's oesophagus (BO) (van Soest *et al*, 2005; de Jonge *et al*, 2010). BO constitutes a premalignant condition leading to OAC. It is characterised by a metaplastic change of the oesophageal squamous mucosa into columnar-lined epithelium, with or without specialised intestinal metaplasia, and caused by chronic gastro-oesophageal reflux (GORD) (Pace *et al*, 2007; Sharma *et al*, 2009). Various cohort studies have reported wide ranges in the incidence rates of OAC in BO patients (Shaheen *et al*, 2000). After excluding both prevalent cancers and high-grade dysplasia (HGD), a recent systematic review and meta-analysis of OAC and HGD incidence rates in BO patients, arrived at an incidence of OAC and HGD combined of 10.2/1000 person years (Sikkema *et al*, 2010). The incidence of OAC alone was 6.3/1000 person years and the incidence of HGD alone was found to be 4.0/1000 person years (Sikkema *et al*, 2010).

The reported wide ranges of observed intervals between BO diagnosis and incident OAC may either be due to large individual differences in progression rates or to large differences in the stage of diagnoses of BO. As practically no reliable long-term predictors of malignant progression have been identified, there is usually no indication at diagnosis which BO patients will survive to incident OAC (Ong *et al*, 2010). On the other hand, studies of large groups of BO patients referred by general practitioners have generated some insights into the patterns of BO incidence, such as its age-specific increase and the delayed BO onset of about 20 years in females (van Blankenstein *et al*, 2005; van Soest *et al*, 2005; Derakhshan and McColl, 2009).

There is by definition an interval between the onset of BO and incident OAC, which we will term the incubation period. In 1992 Cameron and Lomboy estimated that BO developed at a mean age of 40 years, whereas the mean age at OAC diagnosis was 64 years. They concluded from this observation that the average incubation period from BO to OAC was more than 20 years (Cameron and Lomboy, 1992). A mathematical model based on the observed OAC incidence in a Danish cohort, required all OAC cases to have acquired BO before the age of 45. This also suggested an incubation period of several decades (Van Blankenstein *et al*, 2007a). Unfortunately the unrecorded onset of BO precludes measuring its duration by direct observation. Currently the only available surrogate for the date of BO onset is the date of BO diagnosis.

It is therefore impossible to do more than estimate a minimal incubation period. This might be achieved by examining the length of the intervals between BO diagnosis and incident OAC in very large BO follow-up cohorts and wait until all patients had either developed OAC or died from unrelated causes. However, such cohorts are as yet non-existent. In the current study we have now employed the Rotterdam BO follow-up cohort as the best available alternative. This cohort originally comprised of 166 patients with long-segment BO and has now been followed-up for over 30 years. We updated the outcomes of this cohort 14 years after the previous complete assessment in 1994 (van der Burgh *et al*, 1996). Besides seeking data aiding an estimate of the incubation period, our main objectives were, first, to reassess the incidence rate of OAC in the full cohort, and second to assess the predictive value of the length of BO and the presence of low-grade dysplasia (LGD) at index endoscopy for malignant progression.

## Patients and methods

### Study population

All 166 patients of the original Rotterdam BO cohort were reassessed. This cohort consisted of a selection of patients with BO from our records, meeting given criteria and in whom BO was diagnosed between November 1973 and May 1986. The original inclusion criteria were as follows: BO over at least 3 cm of the full circumference and at least 3 months of follow-up after the first BO diagnosis. Patients developing OAC within 3 months of their first BO diagnosis were excluded (Van der Veen *et al*, 1989). Besides obvious additional exclusion criteria, such as HGD at index endoscopy and loss to follow-up, we now added one new exclusion criterion, non-OAC-related death within 2 years of inclusion. This criterion was based on the consideration that a large proportion of patients had been diagnosed in a university hospital and therefore were likely to have been admitted for more life-threatening conditions than GORD, resulting in a reduced life expectancy, which was in fact observed in the 1996 study (van der Burgh *et al*, 1996). Until 2001 this was a purely observational study and the cohort did not undergo endoscopic surveillance. Patients were only re-endoscoped for symptoms. However, after 2001 a large part of the cohort was entered into an endoscopic surveillance programmes.

### Data collection

#### Clinical follow-up

All available cohort data, including the histology revisions collected in 2001 were used as input (Hage *et al*, 2004). The general practitioners or specialists caring for the 42 patients surviving in 2001 were questioned by mail as to patient's vital status and the presence of signs or symptoms suggestive of oesophageal cancer. Whenever necessary, additional information was obtained by telephone, not only from clinicians, but also from nursing homes, the patients themselves or their relatives. For patients who had died, the date and cause of death were ascertained. For patients who had developed oesophageal cancer, detailed information on histology, treatment and outcome was acquired through the clinicians involved.

#### Endoscopy and histology

Data on the length of BO at index endoscopy were obtained from the original endoscopy reports. Over the years all biopsy samples from BO had been taken with standard biopsy forceps, with additional biopsy samples taken from any observed irregularities.

For a partial reassessment of the cohort, published in 2004 a revision of all available histology of 155 patients had been reviewed by a single expert pathologist (Hage *et al*, 2004). Where available these histology results were used, in their absence we fell back on the original histopathology results. After 2001 a large proportion of the 42 survivors had undergone repeated surveillance endoscopies, of which the date and the results were obtained from the gastroenterologist involved.

### Data analysis

Three events were chosen as primary outcomes: death without HGD/OAC (HGD/OAC), incident HGD/OAC and survival without HGD/OAC. The periods between BO diagnosis and event (BPE) were calculated by outcome group and sex.

### Statistical analysis

The ages at BO diagnosis, length of BPE and age at event were calculated for the three outcome groups and statistically compared with the *χ*^2^-test, Fisher's exact test or the Student's *t*-test where appropriate. Analysis of the time schedule of incidence of HGD/OAC was performed with the Kaplan–Meier survival analysis, testing for statistical significance with the log-rank test and the Cox regression model. All statistical analyses were performed with SPSS (Statistical Package for the Social Sciences version 17, IBM, Somer, NY, USA). Two-sided *P*-values below 0.05 were considered significant.

## Results

### Baseline characteristics

**Figure 1:**
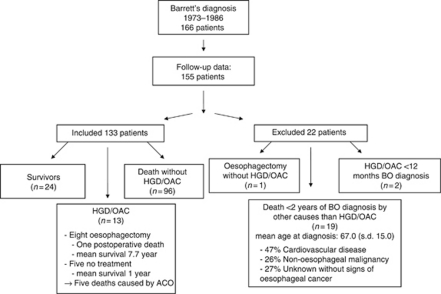
Cohort diagram describing the cohort details, the number of excluded patients, the number of included patients and their outcomes. HGD=high-grade dysplasia; OAC=oesophageal adenocarcinoma.

Thirty-three patients of the original cohort of 166 BO patients were excluded for the following reasons: presence of HGD at baseline (*n*=2), death within 2 years of BO diagnosis (*n*=19), oesophagectomy without HGD/OAC (*n*=1, stricture misclassified as cancer) and loss to follow-up (*n*=11) (Figure 1). The remaining 133 BO patients were included for analysis (M/F 73/60), with an average age at BO diagnosis of 62.4 years (range 14.4–92.3 years), an average BO length of 6.5 cm (range 3–15 cm) and a mean BPE of 14.7 years (range 2.1–32.0 years) (Table 1).

### Differences between sexes

The males in the cohort were significantly younger at BO diagnosis than females (57.4 years *vs* 68.3 years; *P*<0.001). They also had significantly longer BO segments (7.2 cm *vs* 6.0 cm; *P*=0.03) and a longer BPE (16.0 years *vs* 13.3 years; *P*=0.05).

### Outcomes of HGD/OAC

Thirteen patients (M/F 10/3) developed HGD or OAC during follow-up. These were all symptomatic cases of HGD/OAC as the patients were not under endoscopic surveillance and were only reinvestigated for symptoms. These cases were observed over a period of 1967 patient years, 1 per 151 years of follow-up or 0.66 per annum (95% CI 0.58–0.74). Eight patients (M/F 6/2) underwent oesophagectomy, with three postoperative deaths and a mean survival of 8.9 years (range 4.4–15.5) in the survivors. Mean survival in five unoperated patients was 1 year, with two patients dying of OAC and three of co-morbidity. Eventually, in six cases (4.5%) the cause of death was related to HGD/OAC (two directly from OAC, one from metastatic disease 4.4 years after oesophagectomy and three from surgical complications). Two patients who developed HGD/OAC were alive in 2008, their intervals between BO diagnosis and HGD/OAC diagnosis amounted 19.2 and 20.4 years and their current survival post-oesophagectomy was 3.7 and 12.1 years, respectively (Table 2).

### Subjects who died without developing HGD/OAC

Ninety-six patients (72% M/F 44/52) died without HGD/OAC after an average BPE of 12.6 years (range 2.1–26.8). Their mean age at BO diagnosis was 67.4 years (range 29.6–92.3) and the mean age at death was 80.0 years (range 35–100). The mean age at which the males had been diagnosed with BO was 63.5 *vs* 70.7 years in females (*P*<0.001), the male BPE was 12.7 *vs* 12.6 years in females and the ages at event were 76.2 *vs* 83.2 years (*P*<0.0001), respectively (Table 1).

### Survivors without HGD/OAC

Twenty-four patients (18% M/F 19/5, mean age 68.3 years, range 43.7–92.9) were alive on 1 February 2008 without HGD/OAC. In this group there were no differences by sex in mean ages at BO diagnosis, BPE or mean age at event. However, the differences in all these three parameters with the two previous outcome groups were very significant. (Figure 3) Survivors had an average age at inclusion of 42.8 years *vs* 67.4 years in the deceased group; their mean BPE was 25.5 years and the mean age at event was 68.3 years. (Table 1 and Figure 3) The mean BO length was 6.0 cm (range 3–10 cm) in the survivors and 6.4 cm (range 3–15 cm) in the deceased group. This difference in BO length was not responsible for the difference in survival (log rank *P*=0.8) (Table 3).

### Factors predictive of HGD/OAC during follow-up

More males (78.6%) than females developed HGD or OAC. The mean age at BO diagnosis was lower in males, 58.7 (range 37.2–77.8) *vs* 69.7 (range 57.2–76.4) years, but the mean male BPE of 12.2 years (range 3.8–20.4) was far longer than the 6 years in females (range 1.6–10.4) (both *P*-values NS) whereas the mean ages at end point were similar 75.6 (range 58.8–86.5) *vs* 71.5 years (range 56.4–88.7) (Table 1).

No significant differences were demonstrated in age at BO diagnosis between patients developing HGD/OAC and patients who died without developing HGD/OAC. The average BO length was longer in the HGD/OAC group, 8.4 and 6.4 cm in the overall cohort (*P*=0.04) (Table 3).

Twenty-seven (20%) patients were diagnosed with LGD at inclusion. In the absence of endoscopic surveillance until 2001, no data were available about new cases of LGD developing before 2001, in practice no cases were observed after 2001. There were no differences in age at BO diagnosis or follow-up interval between patients with and without LGD at inclusion. However, the length of the BO segment at diagnosis was significantly longer in the patients with LGD, 8.3 *vs* 6.1 cm (*P*=0.001) (Table 4).

**Figure 2:**
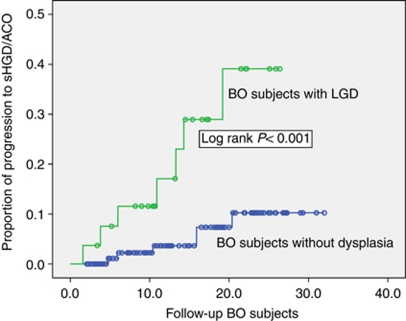
Kaplan–Meier curve demonstrating a difference in progression to HGD/OAC in subjects with BO with or without LGD. BO=Barrett's oesophagus; HGD=high-grade dysplasia; LGD=low-grade dysplasia; OAC=oesophageal adenocarcinoma.

The baseline presence of LGD was associated with progression to HGD/OAC (Log rank *P*<0.001) (Figure 2); 26% of patients with baseline LGD developed HGD/OAC compared with 5.6% in the group without baseline LGD (Table 4). A BO length >8 cm was in univariate analysis also associated with the progression to HGD/OAC (*P*=0.04) (8.3 *vs* 6.3 cm); however, using a Cox-regression model the only factor significantly associated with progression was the presence of LGD at inclusion (*P*=0.004).

## Discussion

The Rotterdam BO cohort, ranging back to 1973, is to our knowledge the longest running observational cohort of long-segment BO patients. The follow-up results of this cohort were previously published in 1989 and 1996 (Van der Veen *et al*, 1989; van der Burgh *et al*, 1996). An analysis limited to the 105 patients with confirmed IM at baseline was published in 2004 (Hage *et al*, 2004). On the basis of current BSG guidelines and United States literature, we have now abandoned this entry criterion of confirmed IM (Playford, 2006; Riddell and Odze, 2009). This is therefore the third follow-up report of the full cohort. Since this study is a continuation of the previous studies on the full cohort, we chose to adhere to the original criterion and excluded prevalent HGD/OAC cases with a diagnosis within 3 months of BO diagnosis. Even though adoption of 1-year interval for prevalent cancers is nowadays more common. This did, however, not affect our results given the fact that no patient was diagnosed with OAC within 1 year after diagnosis of BO.

**Figure 3:**
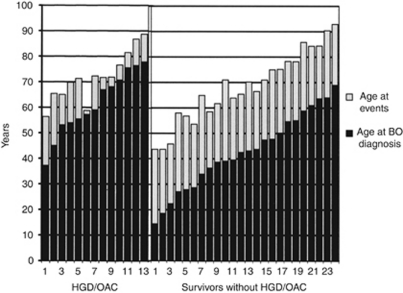
Age at event and age at diagnosis of BO patients developing HGD/OAC and BO patients alive without HGD/OAC. BO=Barrett's oesophagus; LGD=low-grade dysplasia; HGD=high-grade dysplasia; OAC=oesophageal adenocarcinoma. (Event is defined as development of HGD/OAC in the HGD/OAC group and end of follow-up (February 2008) in the survivors).

In our search for the incubation period from BO to HGD/OAC we in particular focused on patients who were diagnosed with BO at a young age. This study revealed the important finding that there is a long incubation period between onset of BO and development of HGD/OAC, as we have demonstrated in Figure 3. In fact in the 24 survivors without HGD/OAC the mean follow-up was 25.5 years, exceeding 30 years in three individuals.

In view of the real date of the onset of BO being unknown, this incubation period is very likely to be in excess of the three decades now observed. The further follow-up of our survivor group will probably extend this observed incubation period even further. The concept of an incubation period is also compatible with the findings in the outcome group of subjects who died without HGD/OAC. The incidence of BO is age related, probably reaching its maximum after age 50 in males and 70 in females (van Blankenstein *et al*, 2005). Although the presence of some cases of asymptomatic HGD/OAC in this outcome group cannot be excluded, the onset of BO apparently occurred too late to bridge the incubation period to incident HGD/OAC. This hypothesis would imply that not only the survivors, but also the HGD/OAC outcome group, should have contracted BO at a relatively early age. This is illustrated in Figure 3, the length of the incubation period was inversely related to the age at BO diagnosis. This supports our hypothesis that the average incubation period is long and that shorter incubation periods in elderly patients are not related to a faster progression, but to a delayed diagnosis of BO. The reason, why, with a few exceptions, the length of the interval to the event in the former group fell far short of our hypothetical several decades, is likely to have been that, at the time when these patients were around 30–40 years of age, upper GI endoscopy, let alone BO diagnosis, was still uncommon. In this respect our survivors were more fortunate in being diagnosed at an earlier age. The recently reported steadily declining age at BO diagnosis, observed between 1990 and 2005, would support this hypothesis (Wall *et al*, 2009). A case report published in 1984 contrasts our hypothesis and describes a short incubation period from Barrett's metaplasia to cancer, however, this case report lacked important information to substantiate this claim (Dahms and Rothstein, 1984).

In our cohort we demonstrated that LGD at inclusion is a clear risk factor for progression to HGD and eventually adenocarcinoma. However the issues of inter and intraobserver variability of dysplasia in Barrett's epithelium have been reported by several studies (Cameron, 1997; Montgomery *et al*, 2001; Conio *et al*, 2003; Dulai *et al*, 2005; Kerkhof *et al*, 2007). Recent large studies including a nationwide Dutch study concluded that, even when taking this variability into account, LGD remains a significant risk factor for progression and can be used as a marker for the need of more intense surveillance (Skacel *et al*, 2000; de Jonge *et al*, 2010).

Another possible risk factor in our cohort was the presence of a BO segment longer than 8 cm at diagnosis, this is, however, a more controversial factor. Although some studies were in line with our observation (Van der Veen *et al*, 1989; Iftikhar *et al*, 1992; Menke-Pluymers *et al*, 1993), others could not demonstrate an association between BO length and progression to HGD (Rudolph *et al*, 2000; Gatenby *et al*, 2007). It might be argued that the significantly greater length of BO and far higher prevalence of LGD in the HGD/OAC group as compared with the survivors and death without HGD/OAC outcome groups could signify a more severe form of BO with a shorter incubation period. The presence of LGD at index endoscopy has been found to indicate a more advanced stage in malignant progression (de Jonge *et al*, 2010). We believe that, in view of the great uncertainty about the true date of onset of BO, the higher prevalence of LGD at baseline in the HGD/OAC group was a sign of more advanced malignant progression than in the other two outcome groups.

The question rises whether the current findings were consistent with the two previous updates published in 1989 and 1996 and, in addition, whether they were compatible with other BO follow-up studies. The incidence rate of HGD/OAC combined was 0.66/100 patient years (95% CI 0.58–0.74), not dissimilar from the 0.59/100 patient years and 0.56/100 patient years OAC incidence observed in the two previous studies, in which all cases later reclassified as HGD were scored as OAC (Van der Veen *et al*, 1989; van der Burgh *et al*, 1996). The results were also compatible with a recent meta-analysis of the OAC risk in BO patients, which arrived at a pooled estimate for the incidence of OAC of 0.62/100 person years (Sikkema *et al*, 2010). Our study was, until 2001, an observational study, so all endoscopies diagnostic for HGD/OAC were performed for symptoms. These symptoms ranged from dysphagia, increasing heartburn or other reflux symptoms. The fact that HGD can cause dysphagia is not always recognised (Gatenby *et al*, 2009). It is interesting to note that the only patient diagnosed with OAC after 2001 became symptomatic during the interval between two surveillance endoscopies.

Should our hypothesis about the protracted latent period between BO onset and OAC incidence be correct, then patients at highest risk of developing OAC would be those with a BO onset at a relatively early age. For efficient endoscopic surveillance they should be identified shortly after BO onset, which would require population screening for BO in the male 40-year age group. This would, however, appear to be a counsel of perfection, although an acceptable non-endoscopic technique was recently proposed (Lao-Sirieix *et al*, 2009). In practice general practitioners could be encouraged to refer male patients with reflux symptoms for endoscopy before prescribing PPI's. This would not only save money now spent on unnecessary PPI consumption but might also improve, if only to a limited extent, the ascertainment of BO at an earlier age (Van Soest *et al*, 2006, 2008).

In conclusion, this third update of the Rotterdam long BO segment observational cohort, (i.e., follow-up without endoscopic surveillance), now spanning over 30 years, produced HGD/OAC incidences consistent with the two previous surveys. However, the most relevant finding was the length of the interval between BO diagnosis and the end of follow-up in the survivors without the occurrence of HGD/OAC. In these survivors, in whom BO was diagnosed at a 20 year earlier mean age than in the rest of the cohort, this interval amounted to a mean of 25.5 years, with three individuals exceeding 30 years. We interpret this observation as indicating an incubation period between the onset of BO and incident OAC of three or more decades. The combination of the age-specific incidence of BO and such a protracted incubation period may well explain why the great majority of BO patients do not contract OAC.

## Figures and Tables

**Table 1:** Characteristics of total cohort; deceased subjects without HGD/OAC, subjects having developed HGD/OAC and survivors without HGD/OAC

**Outcome category**	***N***	**Age at BO diagnosis mean (range)**	**BPE mean (range)**	**Age at end point mean (range)**
*Total cohort*	133	62.4 (14.4–92.3)	14.7 (1.6–32.0)	77.2 (35.0–100.0)
Males	73	57.4 (14.4–84.3)	16.0 (2.3–32.0)	73.5 (35.0–98.4)
Females	60	68.3 (27.0–92.3)	13.3 (1.6–31)	81.6 (48.3–100.0)
				
*Died without HGD/OAC*	96	67.4 (29.6–92.3)	12.6 (2.1–26.8)	80.0 (35.0–100.0)
Males	44	63.5 (32.7–84.3)	12.7 (2.6–25.9)	76.2 (35.0–98.4)
Females	52	70.7 (29.6–92.3)	12.6 (2.1–26.8)	83.2 (48.3–100.0)
HGD/OAC	13	61.2 (37.2–77.8)	10.8 (1.6–20.4)	72.5 (56.4–88.7)
Males	10	58.7 (37.2–77.8)	12.2 (3.8–20.4)	71.5 (56.4–88.7)
Females	3	69.7 (57.2–76.4)	6.0 (1.6–10.4)	75.6 (58.8–86.5)
				
*Survivors without HGD/OAC*	24	42.8 (14.4–68.9)	25.5 (20.8–32.0)	68.3 (43.7–92.9)
Males	19	42.7 (14.4–68.9)	25.6 (22.2–32.0)	68.2 (43.7–92.9)
Females	5	43.1 (27.0–63.6)	25.5 (20.8–31.0)	68.6 (56.8–84.4)

Abbreviations: BO=Barrett's oesophagus; BPE=period between Barrett's diagnosis and endpoint; endpoint=development of HGD/OAC or death or end of follow-up (February 2008); HGD=high-grade dysplasia; OAC=oesophageal adenocarcinoma.

**Table 2:** Outcomes in patients with HGD/OAC

**Patient**	**Treatment**	**Interval BO diagnosis to HGD/OAC (years)**	**Survival after HGD/OAC diagnosis (years)**	**Cause of death**
1	Oesophagectomy	10.4	0.3	OAC
2	Oesophagectomy	4.8	0.2	OAC
3	Oesophagectomy	6.6	15.5	Myocardial infarction
4	No treatment	2.3	3.9	Myocardial infarction
5	Oesophagectomy	14.3	0	OAC
6	Radiation therapy	15.9	0.2	OAC
7	Oesophagectomy	20.4	3.7	Not applicable; alive
8	Oesophagectomy	1.6	4.4	OAC
9	No treatment	10.9	9.3	Heart failure
10	Oesophagectomy	19.2	7.9	Not applicable; alive
11	No treatment	5.9	0.1	Myocardial infarction
12	Oesophagectomy	13.3	13	No oesophageal carcinoma
13	No treatment	15.4	2.4	No oesophageal carcinoma

Abbreviations: BO=Barrett's oesophagus; HGD/OAC=high-grade dysplasia/oesophageal adenocarcinoma; survival=period between diagnosis of HGD/OAC and death or February 2008.

**Table 3:** Length of BO in different subject groups

**Groups**	**Length BO mean (range) (cm)**
Total Cohort	6.0 (3–15)
Died without HGD/OAC	6.4 (3–15)
HGD/OAC	8.3 (3–14)
Survivors without HGD/OAC	6.0 (3–10)

Abbreviations: BO=Barrett's oesophagus; HGD/OAC=high-grade dysplasia or oesophageal adenocarcinoma.

**Table 4:** Differences between subjects with LGD at baseline and patients without LGD

	**No LGD (s.d.)**	**LGD (s.d** **.)**	***P*-value**
Mean age at BO diagnosis	61.59 years (15.97)	65.36 years (12.54)	0.26
Length BO at diagnosis	6.08 cm (2.89)	8.26 cm (3.45)	0.001
BPE	15.03 years (8.26)	13.84 years (7.07)	0.49

Abbreviations: BO=Barrett's oesophagus; BPE=period between Barrett's diagnosis and endpoint; endpoint=development of high-grade dysplasia/oesophageal adenocarcinoma or death or end of follow-up (February 2008); LGD=low-grade dysplasia.
